# The critical insight of family caregivers of individuals with intellectual and developmental disability and severe self‐injurious behavior

**DOI:** 10.1111/dmcn.16212

**Published:** 2024-12-09

**Authors:** Caroline Roberts, Frank Symons

**Affiliations:** ^1^ University of Minnesota Twin Cities – Institute on Community Integration Minneapolis MN USA; ^2^ University of Minnesota – Educational Psychology Minneapolis MN USA

## Abstract

**This commentary is on the original article by Breitbart et al. on pages** 779–787 **of this issue.**

Caring for a child with self‐injurious behavior (SIB) is a significant challenge, which is compounded by the lack of accessible resources to support families coping with severe SIB in autism spectrum disorder (ASD) and other intellectual and developmental disabilities (IDD). As Brietbart et al. discuss, the impact of SIB can be wide‐ranging, profoundly influencing an individual's long‐term health, their family, school environment, and community access.^1^ Although treatment options exist, including behavioral interventions,^2^ there is evidence of a disturbing gap between individuals who need treatment for SIB and those who receive it.^3^ Firsthand accounts of the lived experience of SIB offer crucial insights into why this treatment gap exists and how to close it.

Brietbart et al.'s work offers potential explanations for why some caregivers may hesitate to seek support: ‘caregivers often experience significant stigma around having a child who can exhibit extreme behaviors,’ or ‘families expressed fear of placing their child in residential care as the only available option’. In a similar interview approach with family caregivers, we developed a working model of treatment pathways to SIB suggesting numerous potential barriers during and after an initial consultation with a primary healthcare provider, including knowledge gaps, practical constraints such as short appointment times, differing beliefs about the nature of SIB, and a tendency to wait until crisis.^4^ Both studies converged by concluding that accessing treatment for SIB requires persistent and ongoing work on the part of the caregiver, who is also trying to cope with dangerous behaviors (parent as comprehensive healthcare team;^1^ caregiver‐driven^4^).

Further, SIB impact on the entire family was replicated by inductive analyses across studies.^1^, ^4^ A family systems lens runs somewhat contrary to most treatment approaches to SIB, at least in IDD, in which the treatment is targeted on the individual level. There are no evidence‐based family therapy interventions for SIB in IDD,^5^ yet the caregivers interviewed in the USA and Canada emphasized the importance of family‐level issues like intra‐familial conflict, caregiver readiness to implement more intensive treatments, other sources of family stress, and sibling impacts.^1^, ^4^

Overall, family caregivers can and do offer critical insights for researchers and clinicians: the current approach to SIB treatment in IDD is insufficient on a number of levels. Treatment access is a highly complex and unreliable process that excludes many families from effective care. Although significant progress has been made over the last four decades on the technical side of SIB – risk factors, behavioral mechanisms, prevalence patterns^2^ – current models and interventions do not address the needs of the whole family and many complex, collective action problems remain. As one example, primary healthcare providers (many caregivers' first point of contact) are insufficiently trained to work with patients with IDD and report they feel they are ‘operating without a map’.^6^ The combined and coordinated action of researchers, clinicians, families, and communities will be required to rethink SIB as a collective action problem to be solved; it is the lived experience of those on the frontlines of daily life with SIB in IDD – family caregivers – who hold the most critical insight.

## Data Availability

Not required.
